# Selective inhibitors of a PAF biosynthetic enzyme lysophosphatidylcholine acyltransferase 2[S]

**DOI:** 10.1194/jlr.M049205

**Published:** 2014-07

**Authors:** Megumi Tarui, Hideo Shindou, Kazuo Kumagai, Ryo Morimoto, Takeshi Harayama, Tomomi Hashidate, Hirotatsu Kojima, Takayoshi Okabe, Tetsuo Nagano, Takahide Nagase, Takao Shimizu

**Affiliations:** * Department of Lipid Signaling, Research Institute, National Center for Global Health and Medicine, Shinjuku-ku, Tokyo 162-8655, Japan; † Department of Respiratory Medicine, Faculty of Medicine, University of Tokyo, Bunkyo-ku, Tokyo 113-0033, Japan; ** Open Innovation Center for Drug Discovery, University of Tokyo, Bunkyo-ku, Tokyo 113-0033, Japan; †† Department of Biochemistry and Molecular Biology, Faculty of Medicine, University of Tokyo, Bunkyo-ku, Tokyo 113-0033, Japan; § Core Research for Evolutional Science and Technology (CREST), Japan Science and Technology Agency, Kawaguchi, Saitama 332-0012, Japan

**Keywords:** TSI-01, LPCAT2 inhibitor, *N*-phenylmaleimide derivatives, platelet-activating factor, high-throughput screening, lyso-PAF acetyltransferase, lipid mediator, inflammation, fluorescent probe, lysophospholipid acyltransferase

## Abstract

Platelet-activating factor (PAF) is a potent pro-inflammatory phospholipid mediator. In response to extracellular stimuli, PAF is rapidly biosynthesized by lyso-PAF acetyltransferase (lyso-PAFAT). Previously, we identified two types of lyso-PAFATs: lysophosphatidylcholine acyltransferase (LPCAT)1, mostly expressed in the lungs where it produces PAF and dipalmitoyl-phosphatidylcholine essential for respiration, and LPCAT2, which biosynthesizes PAF and phosphatidylcholine (PC) in the inflammatory cells. Under inflammatory conditions, LPCAT2, but not LPCAT1, is activated and upregulated to produce PAF. Thus, it is important to develop inhibitors specific for LPCAT2 in order to ameliorate PAF-related inflammatory diseases. Here, we report the first identification of LPCAT2-specific inhibitors, *N*-phenylmaleimide derivatives, selected from a 174,000-compound library using fluorescence-based high-throughput screening followed by the evaluation of the effects on LPCAT1 and LPCAT2 activities, cell viability, and cellular PAF production. Selected compounds competed with acetyl-CoA for the inhibition of LPCAT2 lyso-PAFAT activity and suppressed PAF biosynthesis in mouse peritoneal macrophages stimulated with a calcium ionophore. These compounds had low inhibitory effects on LPCAT1 activity, indicating that adverse effects on respiratory functions may be avoided. The identified compounds and their derivatives will contribute to the development of novel drugs for PAF-related diseases and facilitate the analysis of LPCAT2 functions in phospholipid metabolism in vivo.

Platelet-activating factor (PAF, 1-*O*-alkyl-2-acetyl-*sn*-glycero-3-phosphocholine) is a potent pro-inflammatory phospholipid mediator that promotes several life-threatening diseases, including anaphylaxis, sepsis, acute respiratory distress syndrome, bronchial asthma, and other inflammatory states through a G-protein-coupled PAF receptor (PAFR) (1–4). Following extracellular stimulation, PAF is rapidly biosynthesized via a remodeling pathway in inflammatory cells such as macrophages and neutrophils (1, 5). In the remodeling pathway, one of the membrane phospholipids, 1-*O*-alkyl-2-acyl-*sn*-glycero-3-phosphocholine (alkyl-PC) is hydrolyzed by phospholipase A_2_s (PLA_2_s) to produce free fatty acids and lyso-PAF (1-*O*-alkyl-2-hydroxy-*sn*-glycero-3-phosphocholine) (4, 6). Acetyl-CoA:lyso-PAF acetyltransferase (lyso-PAFAT) [EC 2.3.1.67] subsequently converts lyso-PAF to PAF (1, 7), which is rapidly degraded to lyso-PAF and acetic acid by PAF acetylhydrolases (PAF-AHs), terminating its effects (8, 9). Alternatively, lyso-PAF is converted back to alkyl-PC by lysophosphatidylcholine (LPC) acyltransferases (LPCATs) [EC 2.3.1.23] or to fatty aldehyde and glycerolphosphocholine by alkylglycerol monooxygenase (10–13).

We previously identified two entities of lyso-PAFAT belonging to the 1-acylglycerol-3-phosphate *O*-acyltransferase (AGPAT) family: a constitutively expressed lyso-PAFAT, LPCAT1, and an inducible lyso-PAFAT, LPCAT2 (10, 11, 14, 15). LPCAT1 is mainly expressed in the lungs and produces PAF and dipalmitoyl-phosphatidylcholine (DPPC), which is a main component of a pulmonary surfactant essential for respiration (15–17). LPCAT2 is mainly expressed in inflammatory cells and also possesses biosynthetic activities for PAF and cellular membrane phosphatidylcholine (PC) (14). After the activation of Toll-like receptor 4, LPCAT2, but not LPCAT1, is activated by phosphorylation within 30 min and upregulated within 16 h to promote PAF production in mouse macrophages (14, 18). Overexpression of LPCAT2 increases PAF levels in RAW264.7 cells in response to extracellular stimuli (13). These reports indicate that LPCAT2 plays an important role in PAF biosynthesis under inflammatory conditions. LPCAT2 represents a potential target enzyme for the development of novel drugs to ameliorate PAF-related diseases. It is also important to avoid the inhibition of LPCAT1, which is essential for respiratory functions.

To date, two series of compounds have been reported as endogenous lyso-PAFAT inhibitors: tea polyphenols, including (−)-epigallocatechin-3-*O*-(3-*O*-methyl)gallate (EGCG), and fumigatin derivatives, which are oxidized forms of a metabolite from *Penicillium* species (19, 20). However, it is not known whether these compounds have selectivity for LPCAT2 over LPCAT1. In addition, although PAFR antagonists have been developed to ameliorate PAF-related diseases, they are not used as therapeutics because of limited efficacy and/or adverse effects (2). Recently, PAFR-independent effects of PAF have been reported: PAF promotes the neuronal migration of granulocytes and the release of a chemokine, RANTES/CCL5 (regulated on activation, normal T cell expressed and secreted/chemokine ligand 5), from human eosinophils without PAFR signal transduction (21, 22). Thus, it is possible that inhibitors of LPCAT2-mediated PAF biosynthesis might be more valuable for therapeutic applications than PAFR antagonists.

In this study, we employed high-throughput screening (HTS) of a 174,000 compound library to identify *N*-phenylmaleimide derivatives that inhibited the activity of LPCAT2. These compounds also inhibited PAF production in LPCAT2-overexpressing RAW264.7 cells and mouse peritoneal macrophages. These seed compounds may represent novel pharmaceutical agents to ameliorate PAF-related diseases without side-effects due to LPCAT1 inhibition.

## MATERIALS AND METHODS

### Materials

The chemical compound library was from Open Innovation Center for Drug Discovery, University of Tokyo (Tokyo, Japan) (23). The library includes 174,131 compounds supplied as 10 or 2 mM solutions in DMSO. After the selection of *N*-phenylmaleimide derivatives, each compound was obtained in powdered form from commercial vendors (see supplementary Methods). Methylcarbamyl-PAF (mcPAF, nonhydrolyzed analog of PAF), 16:0 PAF, 16:0 lyso-PAF, deuterium-labeled (d_4_)-16:0 PAF, d_4_-16:0 lyso-PAF, and d_31_-16:0 lyso-PC were from Cayman Chemical Company (Ann Arbor, MI). DPPC standards were purchased from NOF Corporation (Tokyo, Japan). Palmitoyl-CoA and arachidonoyl-CoA were from Avanti Polar Lipids (Alabaster, AL). Acetyl-CoA, DMSO, chloroform, and LC-MS grade solvents (methanol and acetonitrile) were from Wako (Osaka, Japan). Lipopolysaccharide (LPS) from *Salmonella minnesota* was purchased from Sigma-Aldrich (St. Louis, MO), and A23187 (calcium ionophore) was from Biomol (Plymouth Meeting, PA). Two types of protease inhibitor cocktails (complete and EDTA-free complete) were purchased from Roche Applied Science (Mannheim, Germany).

### Mice

Female C57BL/6N mice were obtained from Clea Japan, Inc. (Tokyo, Japan). Maintenance of the facility and the use of animals were in full compliance with the Ethics Committee for animal experiments of National Center for Global Health and Medicine.

### Cell culture

RAW264.7 wild-type cells, RAW264.7 cells stably expressing mouse LPCAT2 (RAW-mLPCAT2 cells), Chinese hamster ovary (CHO)-S wild-type cells, and CHO-S cells stably expressing PAFR (CHO-S-PAFR cells) were cultured as previously described (18, 24). Thioglycollate-induced mouse peritoneal macrophages were isolated as previously described (18).

### Transfection of CHO-S and CHO-S-PAFR cells

CHO-S and CHO-S-PAFR cells were transfected with 15 μg of the FLAG-tagged mouse LPCAT (mLPCAT)1, human LPCAT (hLPCAT)1, mLPCAT2, or hLPCAT2 expression vectors using 30 μg of Lipofectamine 2000 (Life Technologies, Carlsbad, CA). Twenty-four hours after transfection, the media were changed to DMEM containing 0.1% BSA and cultured for 24 h.

### Preparation of cell lysates

CHO-S cells were scraped into 1 ml of ice-cold buffer A (20 mM Tris-HCl (pH 7.4), 300 mM sucrose, and 1× EDTA-free complete protease inhibitor cocktail) and sonicated three times on ice for 30 s using a probe sonicator (10 watts; Ohtake Works, Tokyo, Japan). CHO-S-PAFR cells were sonicated in buffer A containing 1 mM sodium orthovanadate. After centrifugation for 10 min at 9,000 × *g*, the supernatants were collected and centrifuged at 100,000 × *g* for 1 h at 4°C. The resultant pellets (microsomal proteins) were resuspended in 20 mM Tris-HCl (pH 7.4) and stored at −80°C. Peritoneal macrophages were sonicated in buffer containing 100 mM Tris-HCl (pH 7.4), 300 mM sucrose, 5 mM 2-mercaptoethanol, and 1× complete protease inhibitor cocktail. After centrifugation, the supernatants (soluble proteins) were also stored at −80°C. Protein concentration was measured using the Bradford protein assay reagent (Bio-Rad, Hercules, CA) and BSA (fraction V, fatty acid-free; Sigma-Aldrich) as a standard.

### Western blot analysis

Western blotting was performed as described previously (18). Cell extracts were separated by SDS-PAGE or Phos-tag SDS-PAGE (NARD Institute, Ltd., Hyogo, Japan) and analyzed by blotting with anti-FLAG M2 antibody (3:1,000) (Sigma-Aldrich).

### HTS

**Fig. 1.**
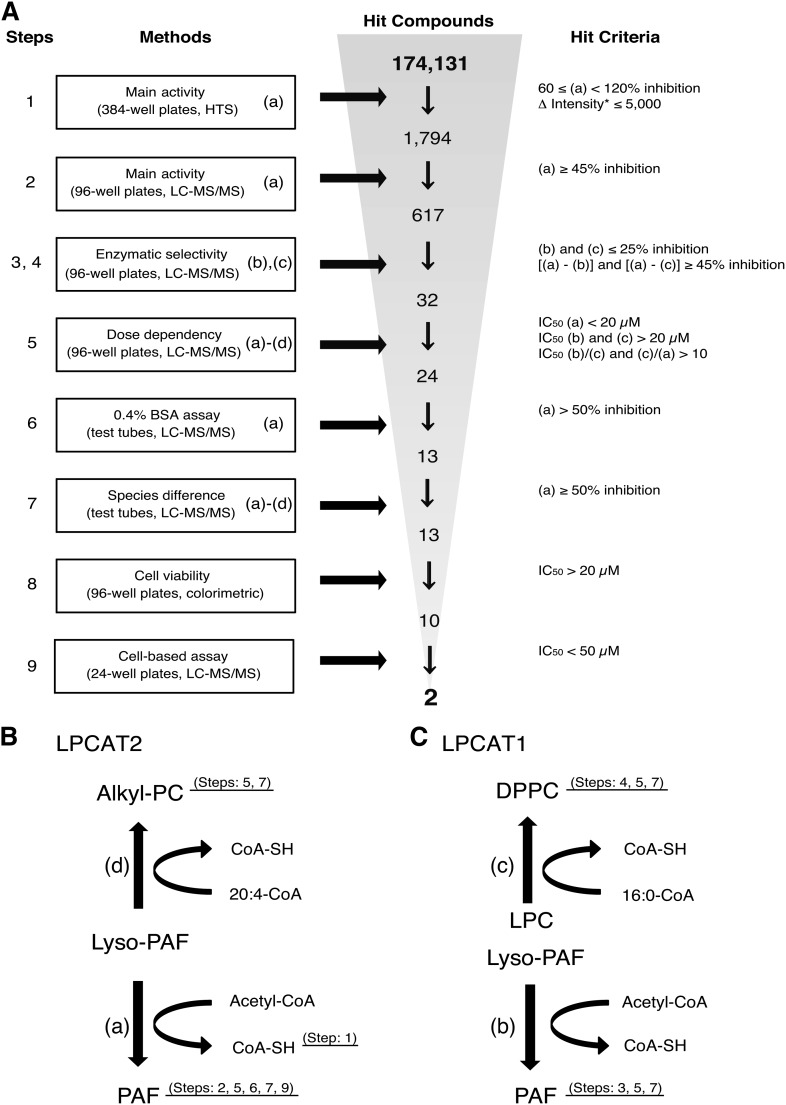
Identification of LPCAT2-specific inhibitors via HTS. A: Screening cascade to identify LPCAT2-specific inhibitors. For each assay, 20 μM of each compound was used. Hit compounds were determined by the indicated criteria. CoA-SH was detected by a fluorescent probe in step 1 (HTS). PAF, DPPC, and 16:0/20:4 alkyl-PC were measured by LC-MS/MS in the following steps. Human enzymes were used in steps 1–6. Both human and mouse enzymes were used in step 7. Two compounds were identified as potent and selective inhibitors of LPCAT2 in the 9-step selection process. *The increased value of inflorescence intensity elicited by the thiol-reactive CPM. Screening steps 1–5 (n = 1), steps 6 and 7 (n = 3), steps 8 and 9 (n = 2). The details are described in the supplementary Methods. B, C: Schematic presentation of the acetyltransferase and acyltransferase activities of LPCAT2 and LPCAT1. Both enzymes have lyso-PAFAT activity [(a) and (b)] and long chain acyltransferase activity [(c) and (d)].

For HTS, 384-well plates were predispensed with 60 nl (2 mM) of each compound. A profluorescent thiol-reactive coumarin maleimide derivative 7-diethylamino-3-(4′-maleimidylphenyl)-4-methylcoumarin (CPM; Life Technologies), was used to detect thiol-containing CoA (25) released in the lyso-PAFAT reaction (Fig. 1B). Microsomal proteins (3 μl, 15 μg/ml) of hLPCAT2-transfected CHO-S-PAFR cells, stimulated with 200 nM mcPAF for 30 s, were added to each well and incubated for 30 min at room temperature with 3 μl of buffer B (100 mM Tris-HCl (pH 7.4), 1 μM CaCl_2_, and 0.0075% Tween-20) and two substrates: 5 μM lyso-PAF (non-labeled) and 25 μM acetyl-CoA. The reaction was terminated with 6 μl of 50% methanol containing 5 μM CPM, and fluorescence intensity (λ_ex_ = 350 nm, λ_em_ = 450 nm) was measured using a PHERAstar microplate reader (BMG LABTECH, Offenburg, Germany) 2 h later and percentage inhibition was calculated. The increases in fluorescence intensity were also evaluated for the compounds with intrinsic fluorescence to determine the change in intensity (Δ intensity). The data were normalized to each positive control set at 100% activation. The assay performance was consistent across all plates, with robust Z′ factors (25). Hit criteria are shown in Fig. 1A.

### Acetyltransferase and acyltransferase assays

Microsomal protein fractions (0.5 μg) supplemented with the indicated compound concentrations were added to the reaction mixture containing 100 mM Tris-HCl (pH 7.4), 1 μM CaCl_2_, 0.015% Tween-20, and two substrates. For acetyltransferase activity, the substrates were 1 mM acetyl-CoA and 5 μM d_4_-lyso-PAF; for the acyltransferase activity of LPCAT1 or LPCAT2, the substrates were, respectively, palmitoyl-CoA and d_31_-16:0 LPC or arachidonoyl-CoA and d_4_-lyso-PAF (all 5 μM). Each lysophospholipid contained fatty acid at the *sn*-1 position. After incubation at 37°C for 5 min, the reactions were stopped by adding 300 μl methanol containing 17:0 LPC or 14:0/14:0 PC as an internal standard. Deuterium-labeled products were analyzed by LC-MS/MS (Waters, Milford, MA and Thermo Scientific, Waltham, MA).

### PAF-AH and PLA_2_ assays

PAF-AH and PLA_2_ activities were determined as previously described (3). Briefly, a soluble protein fraction of peritoneal macrophages was incubated with 20 μM of each compound at 37°C for 30 min in a total volume of 0.1 ml. For the PAF-AH assay, the reaction mixture contained 2.5 μg protein, 50 mM Tris-HCl (pH 7.4), 5 mM EDTA, 5 mM 2-mercaptoethanol, and 1 μM d_4_-PAF. For the PLA_2_ assay, 10 μg protein was incubated in buffer containing 100 mM Tris-HCl (pH 7.4), 1 mg/ml BSA, 4 mM CaCl_2_, 1 mM DTT, and 2 μM 16:0/20:4 alkyl-PC. A cytosolic PLA_2_ inhibitor, pyrrophenone (Sigma-Aldrich), was used as a positive control. The reactions were stopped with 0.27 ml of chloroform/methanol (1:2, v/v), followed by the sequential addition of an internal standard (17:0 LPC or d_4_-lyso-PAF), 0.11 ml chloroform, and 0.1 ml water. Lipids were extracted using the Bligh and Dyer method (26). The PAF-AH and PLA_2_ activities were measured by detecting d_4_-lyso-PAF and nonlabeled lyso-PAF, respectively, using LC-MS/MS.

### Cell viability assay

Cell viability was measured using a modified MTT [3-(4,5-dimethylthiazol-2-yl)-2,5-diphenyltetrazolium bromide] dye reduction assay using WST-8 [2-(2-methoxy-4-nitrophenyl)-3-(4-nitrophenyl)-5-(2,4-disulfophenyl)-2H-tetrazolium, monosodium salt; Dojindo Molecular Technologies, Inc., Kumamoto, Japan] according to the manufacturer’s instructions. RAW267.4 or RAW-mLPCAT2 cells were seeded in 96-well plates (1 × 10^4^ cells/well) for 8 h and then treated with a compound for 36 h. WST-8 was added for another 2 h and the 450 nm absorbance was measured using a microplate reader (Bio-Rad). Cell viability was expressed as a percentage of vehicle-treated control values. To observe the effects of the incubation time on cell viability, 0.4 × 10^4^ RAW-mLPCAT2 cells were treated with compounds for 1 or 6 h before the WST-8 assay.

### Cell-based assay

RAW-mLPCAT2 cells and peritoneal macrophages were seeded in 24-well plates (4 × 10^6^ cells/well) for 24 or 2 h, respectively, before treatment. RAW-mLPCAT2 cells were stimulated with 5 μM A23187 for 5 min after a 1 h treatment with the compounds. Peritoneal macrophages were preincubated with 100 ng/ml LPS for 18 h, treated with the compounds for 1 h, and stimulated with 5 μM A23187 for 5 min. To analyze cellular PAF levels, lipids were extracted with 500 μl methanol containing d_4_-PAF and d_4_-lyso-PAF as internal standards. Each sample was submitted to solid phase extraction using an Oasis HLB 96-well cartridge cluster (Waters), and PAF and lyso-PAF were measured by LC-MS/MS as previously described (13).

### Analysis by LC-MS/MS

Chromatography was performed using an Acquity^™^ Ultra-performance LC system (Waters) coupled to a TSQ Vantage triple stage quadrupole mass spectrometer (Thermo Scientific) with a HESI-II electrospray ionization source (13). Phospholipids were separated on BEH C_8_ columns (1.7 μm, 2.1 × 30 mm or 1 × 100 mm; Waters) for the analysis of assay products or the composition of cellular PAF and lyso-PAF, respectively. The mobile phase contained 20 mM aqueous ammonium bicarbonate and acetonitrile. The extracts from peritoneal macrophages were separated on a Kinetex C_8_ column (1.7 μm, 2.1 × 100 mm; Phenomenex, Torrance, CA) by a gradient elution with 0.05% formic acid-water and acetonitrile, using the Nexera X2 system and LCMS-8080 mass spectrometer (Shimadzu, Kyoto, Japan) (13). Lyso-PAF, d_4_-PAF, and d_31_-DPPC were identified by the choline fragment (*m/z* 184) as a product ion of selected reaction monitoring in the positive ion mode. Nonlabeled PAF and d_4_-16:0/20:4 alkyl-PC were determined by fatty acid anions of *sn-*2 composition (*m/z* 59 and 303, respectively) in the negative ion mode. Mass spectra were processed using the Xcalibur 2.0 (Thermo Scientific) or LabSolutions 5.50 (Shimadzu) software. Signal intensities were determined relative to the internal standard or as the percentage of the positive control.

### Statistics

Data statistical analyses were performed by one-way ANOVA for independent or correlated values followed by the Tukey’s post hoc tests; *P* < 0.05 was considered statistically significant. Percentage inhibition was calculated as previously described (25) and the IC_50_ values were determined by linear regression of percentage inhibition values above and below the reference value (50%) from dose-response curves. All statistical calculations were performed using Microsoft Excel 2010 (Microsoft) or Prism4 (GraphPad Software Inc., La Jolla, CA)

## RESULTS

### HTS of 174,131 compounds

**Fig. 2.**
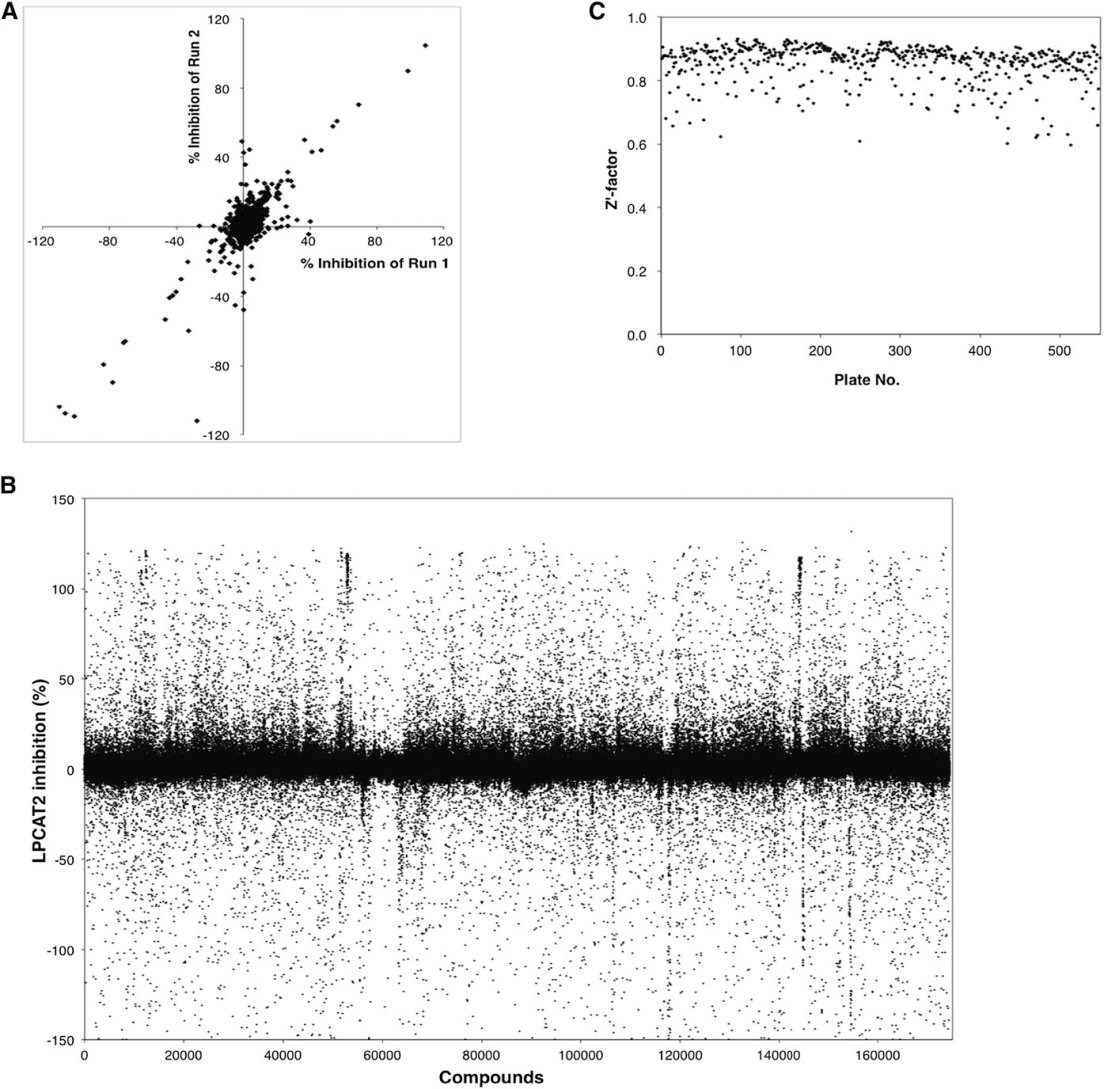
HTS of 174,131 compounds. A, B: Microsomal proteins of hLPCAT2-transfected CHO-S-PAFR cells, stimulated with 200 nM mcPAF, were incubated with lyso-PAF and acetyl-CoA. Thiol-containing CoA was detected by thiol-reactive fluorescent probe, CPM. The data were normalized to each positive control set at 100% activation. Before the library screening, 1,500 compounds were tested and a strong positive correlation was observed (*r* = 0.97) in two independent experiments (runs 1 and 2) (A). Subsequently, 174,131 compounds were analyzed (B). C: The assay performance was consistent across all plates, with robust Z′ factors (>0.5).

To identify specific inhibitors for lyso-PAFAT activity of hLPCAT2, we performed HTS followed by an 8-step hit validation assay using a chemical library of 174,131 compounds from Open Innovation Center for Drug Discovery, University of Tokyo (**Fig. 1A**). In the lyso-PAFAT reaction, PAF and thiol-containing CoA-SH were produced from lyso-PAF and acetyl-CoA, respectively (Fig. 1B, C). In step 1, the inhibitory effects of 20 μM of each compound on lyso-PAFAT activity of hLPCAT2 (reaction (a) in Fig. 1B) were measured by detecting CoA-SH using fluorescent thiol-reactive CPM (**Fig. 2A, B**). We selected 1,794 compounds based on *i*) ≥60% inhibition or *ii*) Δ intensity values ≤5,000 following the lyso-PAFAT reaction (as described in Materials and Methods). The compounds showing ≥120% inhibition were excluded because they were suspected to quench the fluorescence. The assay quality was excellent (Z′ factor = 0.85, Fig. 2C). In step 2, PAF was monitored as a reaction product by LC-MS/MS (a), and 617 compounds were selected based on a ≥45% inhibition. In steps 3 and 4, LPCAT1-produced PAF [reaction (b) in Fig. 1C] and DPPC [reaction (c) in Fig. 1C] were measured, and 32 compounds were selected based on a ≤25% inhibition [in (b) and (c)] or ≥45% inhibition [(a)–(b) and (a)–(c)]. EGCG, one of the lyso-PAFAT inhibitors (19), was eliminated in step 3, because it inhibited the lyso-PAFAT activities of both hLPCAT2 and hLPCAT1 (supplementary Fig. I). In step 5, the IC_50_ for each of the 32 compounds was determined in reactions (a), (b), and (c). Using LC-MS/MS, 24 compounds were selected based on IC_50_ <20 μM in (a) and IC_50_ >20 μM in (b) and (c), or >10 μM in (b)/(a) and (c)/(a). At the 6th step, 11 compounds were excluded, because BSA decreased the inhibitory effects of these compounds (<50% inhibition) on reaction (a). Thus, 13 compounds were selected; these compounds showed inhibitory activity against mouse enzymes as well during step 7 in reactions (a), (b), (c), and (d). Reaction (d) indicated LPCAT2 acyltransferase activity to biosynthesize 16:0/20:4 alkyl-PC (Fig. 1B). In step 8, 10 compounds were selected based on cell viability effects (IC_50_ >20 μM). In step 9, the last step, calcium-ionophore A23187-induced PAF production was measured by LC-MS/MS in RAW-mLPCAT2 cells, and finally two compounds, named TSI-01 (propan-2-yl 4-(3,4-dichloro-2,5-dioxopyrrol-1-yl)benzoate) and TSI-02 (3-chloro-4-morpholin-4-yl-1-(4-phenoxyphenyl)pyrrole-2,5-dione), were found to be active in this cell assay with IC_50_ 28.2 μM and 23.6 μM, respectively. The step-wise selection strategy is summarized in Fig. 1.

### Structure-activity relationship

Because TSI-01 and TSI-02 had the same scaffold, the derivatives of *N*-phenylmaleimide (1-phenylpyrrole-2,5-dione) were examined as candidate inhibitors of LPCAT2 (**Table 1**). For each activity assay, IC_50_ values were determined based on the production of PAF (both hLPCATs), 16:0/20:4 alkyl-PC (hLPCAT2), or DPPC (hLPCAT1). Among the 11 analogs (TSI-01 to TSI-11), TSI-01, -07, -10, and -11 demonstrated approximately 10-fold selectivity for hLPCAT2 over hLPCAT1. These compounds have the same structure except for one moiety at R_2_ or R_4_. TSI-07 contains aminophenol moiety at R_4_, whereas TSI-10 and -11 bear propyl and butyl moieties, respectively, at R_2_. In addition, TSI-10 and -11 inhibited lyso-PAFAT activity of LPCAT2 in vitro with an IC_50_ of approximately 0.1 μM; however, their analog TSI-09 (methyl group at R_2_), similar to TSI-02, showed low inhibitory efficacy and specificity to hLPCAT2. TSI-03 and -04 also showed low inhibitory effects on hLPCAT2 (IC_50_ >10 μM) and no selectivity over hLPCAT1. TSI-05, -06, and -08 did not significantly inhibit lyso-PAFAT activity (IC_50_ >20 μM). Because *N*-ethylmaleimide (NEM), which has a similar structure to *N*-phenylmaleimide, inhibits several enzymes of the AGPAT family (27), we evaluated its inhibitory effects on both enzymes. Although NEM inhibited hLPCAT2 activity more effectively than that of hLPCAT1, its IC_50_ was extremely high (106 μM, Table 1). On the basis of these results, TSI-01, -07, -10, and -11 were selected as candidate LPCAT2-specific inhibitors for further evaluation.

**TABLE 1.**
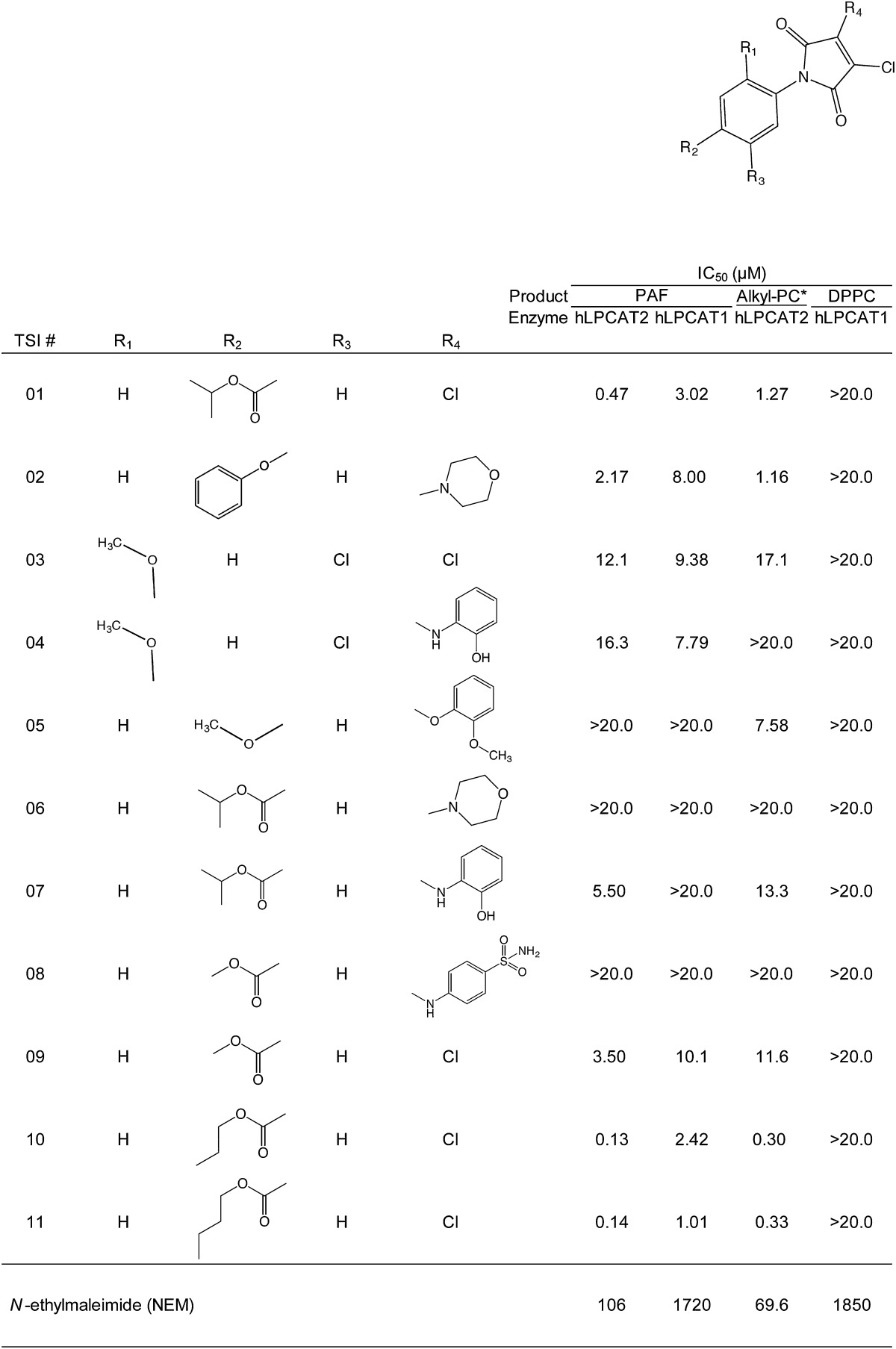
In vitro effects of N-phenylmaleimide derivatives on human LPCAT1 and LPCAT2

Each assay was performed with 0, 0.2, 0.6, 2, 6, and 20 μM of each compound in triplicate. The products were measured using LC-MS/MS. * 16:0/20:4 alkyl-PC.

### Comparison of inhibitory activity against human and mouse enzymes

**Fig. 3.**
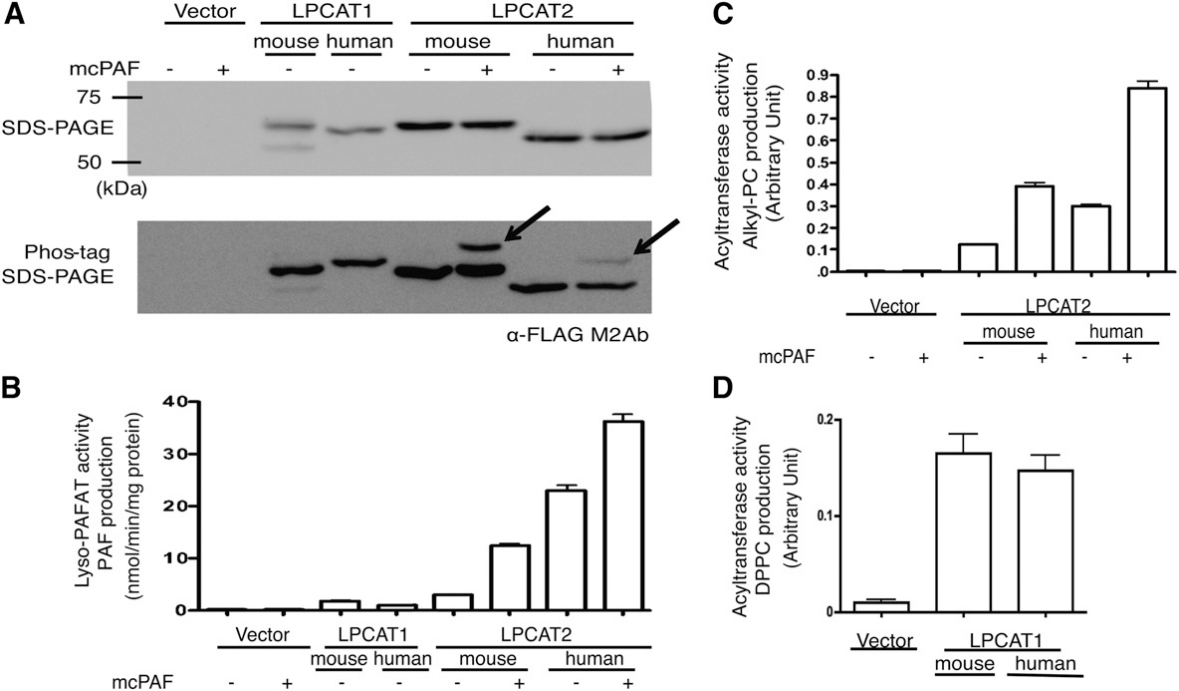
Enzyme expression and activity. A: Microsomal fractions obtained from CHO-S-PAFR cells stimulated or not with mcPAF were separated by SDS-PAGE or Phos-tag SDS-PAGE and analyzed by Western blotting using the anti-FLAG M2 antibody. Upper: protein expression. Lower: on the Phos-tag SDS-PAGE, band shifts (arrow heads) were observed in the stimulated fractions of LPCAT2, indicating phosphorylation upon mcPAF-stimulation. B–D: Both LPCAT acetyltransferase (B) and LPCAT2 acyltransferase (C), or LPCAT1 acyltransferase (D) activities were measured in microsomal fractions; PAF (B), 16:0/20:4 alkyl-PC (C), and DPPC (D) were detected in each assay. The experiment was performed in triplicate (mean ± SEM). Four independent experiments were performed with similar results.

To assess the differences in the inhibitory effects against human and mouse enzymes, we assessed the inhibitory activities of TSI-01, -07, -10, and -11 (20 μM) against hLPCAT and mLPCAT. **Table 2** shows that all tested compounds had similar inhibitory effects on hLPCAT and mLPCAT. Furthermore, when tested in the reaction in which LPCAT2 was activated by mcPAF stimulation (**Fig. 3B, C**), the compounds did not exhibit differences in the inhibition of phosphorylated and unphosphorylated LPCAT2 (data not shown).

**TABLE 2.** Comparison of the effects of compounds on human and mouse enzymes

Product Enzyme	PAF				16:0/20:4 Alkyl-PC		DPPC	
	hLPCAT2	mLPCAT2	hLPCAT1	mLPCAT1	hLPCAT2	mLPCAT2	hLPCAT1	mLPCAT1
TSI-01	99.8 ± 0.1	99.2 ± 0.2	75.9 ± 5.2	89.2 ± 1.8	95.2 ± 1.3	95.5 ± 1.8	25.3 ± 8.7	28.2 ± 4.0
TSI-07	90.8 ± 3.0	84.1 ± 6.4	11.9 ± 13.1	33.6 ± 6.1	83.1 ± 4.3	82.4 ± 5.7	19.5 ± 8.0	36.3 ± 5.7
TSI-10	99.5 ± 0.2	99.1 ± 0.2	76.1 ± 4.4	89.6 ± 1.1	85.4 ± 3.0	86.7 ± 5.2	23.9 ± 3.4	14.8 ± 10.9
TSI-11	99.4 ± 0.2	98.9 ± 0.3	78.6 ± 2.8	89.3 ± 1.1	80.4 ± 2.7	83.0 ± 3.5	14.2 ± 4.8	12.2 ± 20.5

Assays were performed with 20 μM of each compound using the microsomal fractions of nonstimulated CHO-S-PAFR cells transiently transfected with LPCAT2 or LPCAT1. Data are shown as percentage inhibition (n = 3, mean ± SEM).

### Effect on cell viability

To eliminate cytotoxic compounds, we examined cell viability in RAW264.7 and RAW-mLPCAT2 cells using the WST-8 colorimetric assay. All four compounds, TSI-01, -07, -10, and -11, did not show significant cytotoxicity (IC_50_ >20 μM) after a 36 h treatment (data not shown).

### Inhibitory effects of TSI compounds on PAF production in RAW-mLPCAT2 cells

**Fig. 4.**
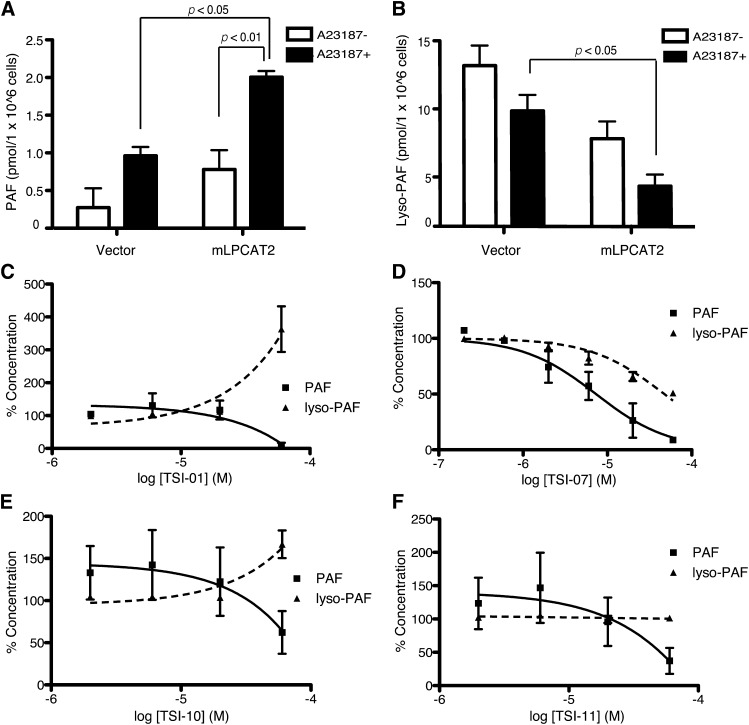
Effects of compounds on RAW-mLPCAT2 cells. A, B: RAW-mLPCAT2 cells and control RAW cells were stimulated with 5 μM A23187 for 5 min and the levels of PAF (A) and lyso-PAF (B) were determined by LC-MS/MS; statistical analyses were performed using ANOVA and Tukey’s multiple comparison test. C–F: RAW-mLPCAT2 cells were treated with the indicated concentrations of TSI-01 (C), TSI-07 (D), TSI-10 (E), or TSI-11 (F) for 1 h before A23187 stimulation, and PAF and lyso-PAF levels were determined by LC-MS/MS. All compounds dose-dependently decreased PAF levels. Data are presented as percentages of the control (0.2% DMSO): 100% PAF and lyso-PAF levels were 1.2 and 5.0 (C), 2.4 and 11.8 (D), or 1.0 and 5.9 (E and F) pmol/1 × 10^6^ cells, respectively. All data are shown as the mean ± SEM of three independent experiments performed in triplicate.

Next, we investigated whether TSI inhibitors suppressed PAF production in RAW-mLPCAT2 cells. Cells were treated with 5 μM A23187 for 5 min, and PAF and lyso-PAF were assessed by LC-MS/MS. In RAW-mLPCAT2 cells, PAF levels were increased and lyso-PAF levels tended to decrease after A23187 stimulation (**Fig. 4A, B**). A23187-induced PAF production was suppressed by 1 h pretreatment with TSI-01, -07, -10, or -11 (Fig. 4C–F) without a decrease in cell viability (IC_50_ >60 μM). Inhibition of LPCAT2 was expected to increase the amount of the PAF precursor; however, lyso-PAF level was decreased by TSI-07 (Fig. 4D) and was not changed by TSI-11 (Fig. 4F). Both TSI-01 and -10 increased lyso-PAF levels, whereas TSI-01 was a more potent inhibitor of PAF production (IC_50_ = 38.8 μM) than was TSI-10 (Fig. 4C, E). Neither PAF-AH nor PLA_2_ activity was decreased in the presence of any of the four TSI compounds (data not shown). These results indicate that TSI-01 was the most potent and selective LPCAT2 inhibitor; therefore, we focused on TSI-01 in our further investigations.

### TSI-01 inhibition of PAF production in mouse peritoneal macrophages

**Fig. 5.**
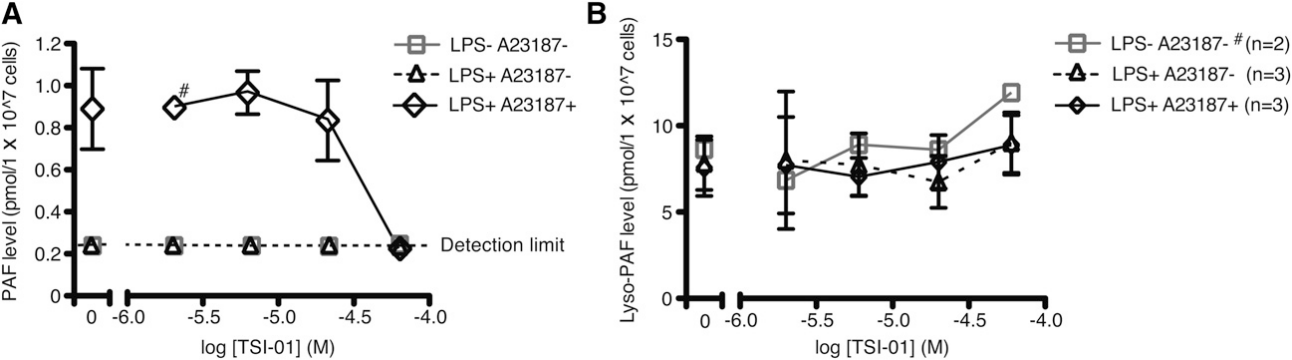
Effects of TSI-01 on PAF and lyso-PAF production in mouse peritoneal macrophages. A, B: Cells were pretreated with LPS for 18 h and then treated with the indicated concentrations of TSI-01 for 1 h, then PAF (A) and lyso-PAF (B) levels were measured after 5 μM A23187 stimulation for 5 min. TSI-01 dose-dependently reduced PAF levels; in the cells treated with 60 μM TSI-01 or not stimulated with A23187, PAF levels were below the detection limit. Lyso-PAF levels were unchanged. Data are shown as the mean ± SEM of three independent experiments performed in triplicate (#, n = 2).

The effect of TSI-01 on PAF and lyso-PAF biosynthesis was evaluated using thioglycollate-elicited mouse peritoneal macrophages. After treatment with 100 ng/ml LPS for 18 h, cells were incubated with TSI-01 for 1 h and subsequently stimulated with 5 μM A23187 for 5 min. PAF levels in A23187-stimulated cells were decreased by TSI-01 pretreatment in a dose-dependent manner (**Fig. 5A**), whereas lyso-PAF levels were not changed by the treatment with LPS, A23187, or TSI-01 (Fig. 5B).

### TSI-01 effects on LPCAT2 activity kinetics

**Fig. 6.**
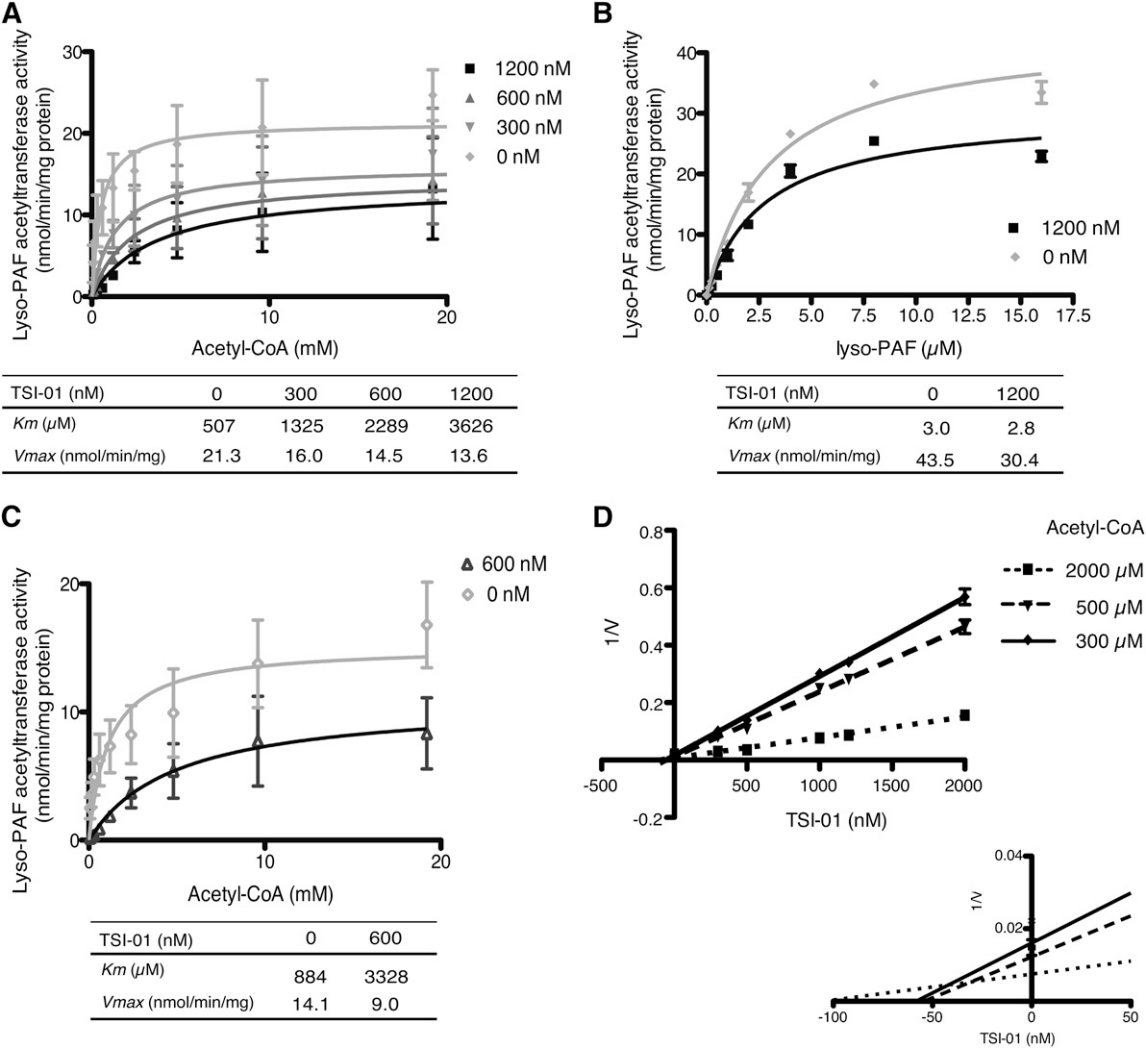
Effects of TSI-01 on kinetics of LPCAT2 enzymatic activity. A, C, D: Lyso-PAFAT activity was measured with 5 μM lyso-PAF and the indicated concentrations of acetyl-CoA using microsomal fractions of mcPAF-stimulated (A, D) or nonstimulated (C) CHO-S-PAFR cells transiently transfected with hLPCAT2; TSI-01 dose-dependently increased *K_m_* values (A, C) and Dixon plot represents the inhibition constant (*K_i_* value) (D). B: Lyso-PAFAT activity was measured with 10 mM acetyl-CoA and the indicated concentrations of lyso-PAF using microsomal fractions of mcPAF-stimulated CHO-S-PAFR cells transiently transfected with hLPCAT2; *K_m_* values were unchanged. The data represent the mean ± SD of triplicate measurements. The experiment was repeated twice with similar results.

To investigate the mechanism by which TSI-01 inhibited lyso-PAFAT activity of hLPCAT2, kinetic analysis was performed. CHO-S-PAFR cells overexpressing hLPCAT2 were stimulated with or without mcPAF for 30 s and analyzed for lyso-PAFAT activity in the microsomal fraction. Apparent *K_m_* values of acetyl-CoA were markedly increased by TSI-01 in both hLPCAT2 stimulated with or without mcPAF (**Fig. 6A, C**); however, those of lyso-PAF were unchanged (Fig. 6B); a slight decrease in *V_max_* values was detected (Fig. 6A–C). Inhibition constant (apparent *K_i_* value) of TSI-01 was also calculated as 30–40 nM using Prism software (Fig. 6D). These results indicate that TSI-01 competitively inhibited the lyso-PAFAT activity of hLPCAT2 with acetyl-CoA.

## DISCUSSION

This is the first report on LPCAT2-specific inhibitors. We screened a library containing 174,131 compounds using a high-throughput fluorescence-based assay, followed by LC-MS/MS-based secondary analysis with excellent qualities and high sensitivities. Among the compounds, *N*-phenylmaleimide derivatives were identified as potential candidate inhibitors. One of these derivatives, TSI-01, was a potent inhibitor against both human and mouse LPCAT2 and competitively blocked the lyso-PAFAT activity with acetyl-CoA. The compound indeed suppressed PAF production in macrophages.

Recently, EGCG and fumigatin derivatives have been reported to decrease endogenous lyso-PAFAT activity in the microsomal fraction of rat spleen; however, the specificity of these compounds for LPCAT1 and LPCAT2 has not been examined (19, 20). EGCG was shown to exert its effects on several molecules, such as cyclooxygenase 2 via the 67 kDa laminin receptor (28). In this study, we confirmed that EGCG strongly inhibited the lyso-PAFAT activity of both LPCAT1 and LPCAT2 (supplementary Fig. I), and showed that TSI-01 and its derivatives had a higher preference for LPCAT2 than for LPCAT1 (Table 1). Previous studies have suggested that LPCAT1 is an important factor required for visual functions through stabilization of retinal photoreceptors and respiratory functions via the production of a pulmonary surfactant (15, 29–31). Thus, LPCAT2-specific TSI compounds should not cause adverse-effects related to LPCAT1 inhibition in vivo. In addition, TSI inhibitors can be used for investigation of the differential roles of PAF generated by LPCAT1 and LPCAT2.

It has been shown that PAF can elicit species-specific eosinophil responses that are independent of PAFR activation to PAF (22); these could be reasons why PAFR antagonists have not shown efficacy in human clinical trials. Inhibition of lyso-PAFAT may prevent biological effects of PAF in both PAFR-dependent and -independent pathways. Therefore, TSI compounds can present a platform for the development of novel therapeutic agents against PAF-related diseases. PAF has also shown anti-obese effects in PAFR-deficient mice (32, 33) and the mRNA of LPCAT2 was upregulated in brown adipose tissue (33). Although it is possible that inhibition of LPCAT2 may promote obesity, our compound may be a valuable reagent to investigate the role of LPCAT2 in obesity in vivo. Further experiments, including pharmacokinetic studies are needed to clarify the roles of LPCAT2 in vivo and the effects of TSI inhibitors.

A number of extracellular stimuli enhance not only acetyltransferase but also the acyltransferase activity of LPCAT2 (Fig. 3) (14, 18). In the remodeling pathway, calcium-dependent cytosolic PLA_2_α is also activated by pro-inflammatory signals and releases arachidonic acid, an eicosanoid precursor that plays diverse roles in inflammatory and immune responses (4, 6). LPCAT2 recognizes arachidonoyl-CoA as a substrate for alkyl-PC production (10, 14), and thus may contribute to arachidonic acid metabolism and membrane remodeling in cells. However, the physiological and pathological importance of this LPCAT2 activity remains unknown. TSI-01 inhibited LPCAT2 acyltransferase activity (Table 1), but not PLA_2_ activity (data not shown). Therefore, TSI-01 and other TSI derivatives may be valuable tools for the elucidation of LPCAT2 acyltransferase activity in inflammation in both in vitro and in vivo settings.

TSI-01 increased the *K_m_* of acetyl-CoA for the lyso-PAFAT activity of LPCAT2, regardless of the enzyme phosphorylation state (Fig. 6). Our results suggest that TSI-01 may competitively inhibit the activity with acetyl-CoA and react within the AGPAT motif 2 (Gly^176^ to Gln^183^) (11, 14, 34) of LPCAT2, previously characterized as a putative acetyl-CoA binding site (15). NEM inhibited LPCAT2 rather than LPCAT1 in a manner similar to TSI-01 (Table 1), suggesting that the thiol-reactive *N*-maleimide backbone is an essential structure for TSI-01 specificity to LPCAT2 over LPCAT1 (27, 35). On the other hand, the selectivity of TSI derivatives for LPCAT2 was abolished by the 5-chloro-2-methoxyphenyl moiety attached to the *N*-malemide backbone in TSI-03 and -04 (Table 1). Further structure-activity studies, including LPCAT2 cocrystallization with TSI compounds, are important to determine the TSI-01 binding and/or positioning site for LPCAT2 and to optimize the structures of LPCAT2-specific inhibitors. On the basis of the structure-dependent activity and toxicity reported for other *N*-phenylmaleimide derivatives (36), we think that TSI-01 derivatives may have a potential as novel highly specific LPCAT2 inhibitors with low toxicity and high membrane permeability.

In conclusion, TSI-01 and its derivatives are promising novel candidate inhibitors for the treatment of PAF-related diseases. The multi-step screening approach described here may be used for the screening of inhibitors or activators of lysophospholipid acyltransferases in general.

## Supplementary Material

Supplemental Data
