# Supplementary material for: Selective inhibitors of a PAF biosynthetic enzyme lysophosphatidylcholine acyltransferase 2
Source: J Lipid Res. 2014 Jul;55(7):1386–96. doi: 10.1194/jlr.M049205 (PMC4076079; doi:10.1194/jlr.M049205)
Supplement: Supplemental Data [file supp_55_7_1386__index.html]

Selective inhibitors of a PAF biosynthetic enzyme lysophosphatidylcholine acyltransferase 2 — Selective inhibitors of a PAF biosynthetic enzyme lysophosphatidylcholine acyltransferase 2 — Supplemental Data 

# Selective inhibitors of a PAF biosynthetic enzyme lysophosphatidylcholine acyltransferase 2

## Supplemental Data

**Files in this Data Supplement:**

- Supplemental figure 1 - Supplemental figure
- Supplemental method - Supplemental method
