# Supplementary material for: Selective inhibitors of a PAF biosynthetic enzyme lysophosphatidylcholine acyltransferase 2
Source: J Lipid Res. 2014 Jul;55(7):1386–96. doi: 10.1194/jlr.M049205 (PMC4076079; doi:10.1194/jlr.M049205)
Supplement: Supplemental Data [file supp_M049205_jlr.M049205-1.pdf]

S. Fig. 1

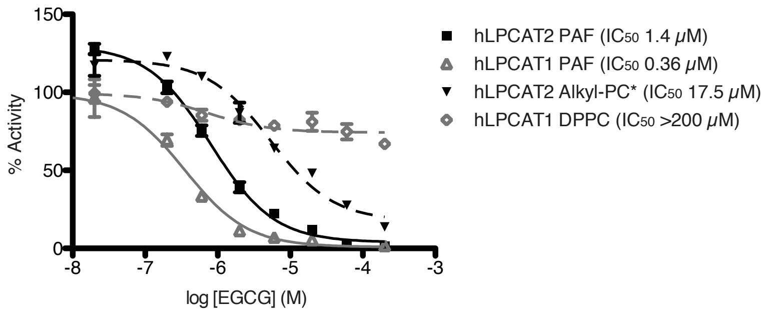

150
100
50
0
% Activity
-8
-7
-6
-5
-4
-3
log [EGCG] (M)
hLPCAT2 PAF (IC50 1.4 μM)
hLPCAT1 PAF (IC50 0.36 μM)
hLPCAT2 Alkyl-PC* (IC50 17.5 μM)
hLPCAT1 DPPC (IC50 >200 μM)

**Supplementary Fig. 1. EGCG effects on enzymatic activity**

Microsomal fractions of CHO-S-PAFR cells were analyzed in acetyltransferase and acyltransferase activity assays performed with the indicated EGCG concentrations. EGCG demonstrated lower $IC_{50}$ for hLPCAT1 than for hLPCAT2 lyso-PAFAT activity. The data represent the mean ± SD of triplicate measurements. Two independent experiments were performed with similar results.
