# Supplementary material for: Selective inhibitors of a PAF biosynthetic enzyme lysophosphatidylcholine acyltransferase 2
Source: J Lipid Res. 2014 Jul;55(7):1386–96. doi: 10.1194/jlr.M049205 (PMC4076079; doi:10.1194/jlr.M049205)
Supplement: Supplemental Data [file supp_M049205_jlr.M049205-2.pdf]

Supplementary Methods

Compounds

The following compounds were obtained from the commercial vendors indicated below and used without further purification.

TSI-01 (propan-2-yl 4-(3,4-dichloro-2,5-dioxopyrrol-1-yl)benzoate, # 7014760174), TSI-03 (3,4-dichloro-1-(5-chloro-2-methoxyphenyl)pyrrole-2,5-dione, # 7010240078), TSI-04 (3-chloro-1-(5-chloro-2-methoxyphenyl)-4-(2-hydroxyanilino)pyrrole-2,5-dione, # 7010670108) were from OTAVA.

TSI-05 (3-chloro-4-(2-methoxyphenoxy)-1-(4-methoxyphenyl)pyrrole-2,5-dione, # 7185200) was from ChemBridge.

TSI-06 (propan-2-yl 4-(3-chloro-4-morpholin-4-yl-2,5-dioxopyrrol-1-yl)benzoate, # F3098-7984) and TSI-07 (propan-2-yl 4-[3-chloro-4-(2-hydroxyanilino)-2,5-dioxopyrrol-1-yl]benzoate, # F3098-7985) were from LifeChemicals.,

TSI-08 (methyl 4-[3-chloro-2,5-dioxo-4-(4-sulfamoylanilino)pyrrol-1-yl]benzoate, # 7015101744) TSI-09 (methyl 4-(3,4-dichloro-2,5-dioxopyrrol-1-yl)benzoate, # 7013910733) TSI-10 (propyl 4-(3,4-dichloro-2,5-dioxopyrrol-1-yl)benzoate, #

7014760173), and TSI-11 (butyl 4-(3,4-dichloro-2,5-dioxopyrrol-1-yl)benzoate, # 7014760172) were from OTAVA.

TSI-02 (3-chloro-4-morpholin-4-yl-1-(4-phenoxyphenyl)pyrrole-2,5-dione) was synthesized as follows: To a solution of *N*-phenoxyphenyldichloromaleimide (1) (1.5 g, 4.5 mmol) in 25 ml of dichloromethane was added morpholine (782 mg, 9.0 mmol) in 2 ml of dichloromethane, and stirred for 1 h at room temperature. The reaction mixture was concentrated and separated on a silica gel column chromatography using hexane-ethyl acetate (80:20) to give TSI-02 (155mg, 9.5% yield). LC-MS (ESI) *m/z* 385.4 ([M + H]$^+$); ^{1}H NMR (DMSO-d_6): δ 3.72-3.75 (4H, m), 3.91-3.94 (4H, m), 7.05-7.09 (4H, m), 7.19 (1H, t, J = 7.1 Hz), 7.31 (2H, d, J = 8.7 Hz), 7.40-7.46 (2H, m).

Steps 2 to 5 screening: 96-well plate assay

Acetyltransferase and acyltransferase assays were developed in 96-well plates. The microsomal hLPCAT2, obtained from mcPAF-stimulated CHO-S-PAFR cells was used in steps 1, 2, and 5. The microsomal hLPCAT1 obtained from unstimulated CHO-S cells was used in steps 3 to 5. Each well of the 96-well plates contained 30 μl of 15 μg/ml protein, 600 nl of 2 mM compound, 30 μl buffer C, and two substrates (Fig. 1B and C). Substrate concentrations were as follows: acetyl-CoA, 250 or 1000 μM acetyl-CoA;

arachidonoyl-CoA, 5 μM; palmitoyl-CoA, 5 μM; d_4-lyso-PAF, 5 μM; and d_{31}-LPC, 5 μM. For step 5, compound concentrations were 0, 0.167, 0.67, 2, 6.7, and 20 μM. The deuterium-labeled products were measured by LC-MS/MS. Percentage inhibition (steps 2 to 4) or IC_{50} (step 5) was used as the hit criterion.

Step 6 screening: BSA assay

The acetyltransferase assay was performed with 20 μM of each compound and microsomal hLPCAT2 from step 1. The assay mixture contained 0.4% BSA and two substrates for lyso-PAFAT activity: 1 mM acetyl-CoA and 5 μM d_4-lyso-PAF. Percentage inhibition values were determined.

Step 7 screening: species-specific differences

To evaluate the effects on the activity of mouse or human LPCAT1 and LPCAT2, each microsomal fraction was obtained using CHO-S-PAFR cells with or without mcPAF (200 nM) stimulation for 5 min. Acetyltransferase and acyltransferase assays were performed with 20 μM of each compound. Percentage inhibition was calculated in each assay.

Step 8 screening: cell viability assay

Wild-type RAW264.7 cells and RAW264.7 cells transfected with mLPCAT2 or control plasmid were used. Cells were cultured for 36 h with 0, 0.06, 0.2, 0.6, 2, 6, and 20 μM of compounds in 96-well plates and assayed for viability with WST-8.

Step 9 screening: cell-based assay

RAW-mLPCAT2 cells were treated with compounds for 1 h and analyzed after 5 min stimulation with A23187. The assays were performed with 0, 0.2, 0.6, 20, and 50 or 60 μM of compound. PAF concentration was measured by LC-MS/MS.

Data analysis

The data were normalized to each positive control (100% activation). Percentage inhibition or IC_{50} were determined in each screening step. To assess screening quality, the percentage coefficient of variance and the Z' factor was calculated for each plate or each assay as described previously (2).

References

1. Relles HM & Schluenz RW. 1972. Dichloromaleimide chemistry. I. Substituent effects on carbon-13 nuclear magnetic resonance and mass spectra. *J Org Chem* **37**: 1742–1745.

2. Chung, C. C., K. Ohwaki, J. E. Schneeweis, E. Stec, J. P. Varnerin, P. N. Goudreau, A. Chang, J. Cassaday, L. Yang, T. Yamakawa, O. Kornienko, P. Hodder, J. Inglese, M. Ferrer, B. Strulovici, J. Kusunoki, M. R. Tota, and T. Takagi. 2008. A fluorescence-based thiol quantification assay for ultra-high-throughput screening for inhibitors of coenzyme A production. *Assay Drug Dev Technol* **6**: 361-374.
